# Beyond Disease Categories: The Adaptive Hierarchical Domain Network (AHDN) for Precision Phenotyping and Personalized Management of Chronic Orofacial Pain

**DOI:** 10.3390/clinpract16080154

**Published:** 2026-08-20

**Authors:** Takahiko Nagamine

**Affiliations:** Department of Psychiatric Internal Medicine, Sunlight Brain Research Center, Hofu 7470066, Japan; tnagamine@outlook.com

**Keywords:** adaptive hierarchical domain network, chronic orofacial pain, burning mouth syndrome, precision medicine, biomarkers, network medicine

## Abstract

**Background/Objectives:** Chronic orofacial pain disorders are highly heterogeneous and therapeutically challenging. Although current classifications such as the International Classification of Orofacial Pain (ICOP) and the concept of nociplastic pain have improved diagnostic consistency, patients with the same diagnosis may show substantial differences in symptom severity, underlying biology, and treatment response. This suggests that disease classifications may describe clinical phenotypes more effectively than the biological mechanisms sustaining persistent pain. This article proposes the Adaptive Hierarchical Domain Network (AHDN) as a conceptual framework for understanding chronic orofacial pain from a precision medicine perspective. **Conceptual Framework:** AHDN integrates concepts from systems biology, network neuroscience, predictive processing, biomarker research, and precision medicine. It organizes chronic pain across five hierarchically related but dynamically interacting layers: biological susceptibility, peripheral nociceptive drivers, central adaptive plasticity, behavioral adaptation, and social embedding. The framework is intended to complement, rather than replace, established diagnostic classifications. **Implications:** The proposed framework suggests that burning mouth syndrome, persistent dentoalveolar pain disorder, persistent idiopathic facial pain, occlusal dysesthesia, and subsets of temporomandibular disorders may represent different mechanistic configurations within a shared adaptive network. AHDN provides a hypothesis-generating structure for mechanistic phenotyping, biomarker development, and individualized management. However, the framework remains conceptual and requires prospective clinical validation, standardized assessment methods, and evaluation of its clinical utility before routine implementation.

## 1. Introduction

Chronic orofacial pain represents one of the greatest unmet challenges in contemporary oral medicine. Patients frequently present with persistent oral burning, dentoalveolar pain, facial pain, or occlusal discomfort despite the absence of clinically significant structural abnormalities. These conditions are often associated with sleep disturbance, anxiety, depression, impaired quality of life, and repeated healthcare utilization, yet their biological mechanisms remain incompletely understood [1,2,3,4]. The introduction of the International Classification of Orofacial Pain (ICOP) has greatly improved diagnostic standardization by providing internationally accepted diagnostic criteria for chronic orofacial pain disorders [5]. Similarly, recognition of nociplastic pain has transformed modern pain medicine by acknowledging altered nociceptive processing as an independent mechanism distinct from nociceptive and neuropathic pain [6,7]. Together, these advances have substantially improved clinical communication and research consistency.

Limitations in current classifications arise because patients with identical ICOP diagnoses frequently show remarkably different clinical courses, biological abnormalities, and responses to treatment [8]. Conversely, patients assigned different diagnostic labels often exhibit strikingly similar neurobiological characteristics. Such observations suggest that current classifications successfully define what patients experience, but less effectively explain why different patients develop persistent pain through different biological pathways. This limitation reflects a broader challenge throughout medicine. Increasingly, complex chronic disorders are understood not as diseases caused by single pathological lesions but as emergent conditions arising from interactions among multiple biological systems. Similar conceptual shifts have occurred in oncology, immunology, psychiatry, and neurology, where precision medicine emphasizes mechanistic phenotyping rather than reliance on diagnostic categories alone [9,10].

Recent discoveries further support this perspective in chronic orofacial pain. Genetic susceptibility, epigenetic regulation, gut microbiota, neuroimmune interactions, endocrine modulation, peripheral small-fiber pathology, altered functional brain connectivity, predictive processing, and behavioral adaptation have all been implicated in persistent pain [11,12,13]. However, these mechanisms are usually investigated independently, making it difficult to understand how they collectively generate the diverse clinical phenotypes encountered in daily practice.

A clinically useful framework should therefore accomplish three objectives [14,15]. First, it should integrate existing biological knowledge into a coherent organizational model. Second, it should facilitate precision phenotyping by identifying the dominant mechanisms contributing to persistent pain in individual patients. Third, it should provide practical guidance for biomarker development and personalized therapeutic decision-making.

In this article, we propose the Adaptive Hierarchical Domain Network (AHDN) as an integrative clinical framework that addresses these objectives. Rather than replacing current diagnostic systems, the AHDN framework complements disease classification by organizing chronic orofacial pain into five hierarchically interacting biological layers that continuously adapt throughout disease progression. We further discuss its clinical applications, illustrate its implementation through a practical methodological workflow, and consider its potential role in future precision medicine.

## 2. Why Current Models Need Further Integration

Several conceptual frameworks have significantly advanced the understanding of chronic orofacial pain. The conceptual contribution of AHDN is therefore not the introduction of a new pain mechanism or diagnostic category. Rather, its proposed novelty lies in organizing mechanisms that are often considered separately into a single hierarchical and dynamically interacting framework for mechanistic phenotyping. ICOP provides diagnostic classification; the biopsychosocial model emphasizes the interaction of biological, psychological, and social influences; nociplastic pain describes altered nociceptive processing; and network medicine emphasizes interconnected biological systems. AHDN is intended to complement these approaches by providing a conceptual structure through which their relevant elements can be considered together and related to individualized mechanistic profiles. However, each focuses primarily on a particular aspect of disease biology. The ICOP provides a robust diagnostic classification based on symptom distribution and clinical criteria, but it does not explicitly describe the biological interactions responsible for symptom persistence [16]. The biopsychosocial model successfully recognizes the importance of biological, psychological, and social influences but generally presents these domains as parallel contributors without defining their hierarchical relationships [17]. Likewise, the concept of nociplastic pain emphasizes altered central nociceptive processing but does not fully integrate upstream biological susceptibility or downstream behavioral and environmental adaptation [18].

Recent advances in systems biology and network neuroscience increasingly suggest that chronic diseases emerge through interactions among dynamically interconnected biological systems rather than isolated pathological mechanisms [19,20]. Similarly, precision medicine has shifted attention toward identifying mechanistic subtypes within apparently homogeneous diagnostic categories [21]. These developments indicate the need for an integrative clinical framework capable of linking molecular biology, peripheral pathology, neural network adaptation, behavioral responses, and environmental influences into a unified conceptual model. Such a framework should support both mechanistic understanding and clinical decision-making while remaining sufficiently flexible to incorporate future biomarkers and emerging therapeutic strategies. The Adaptive Hierarchical Domain Network (AHDN), presented below, is proposed as one possible framework to achieve these objectives.

## 3. The Adaptive Hierarchical Domain Network (AHDN): A New Clinical Framework

**Figure 1:**
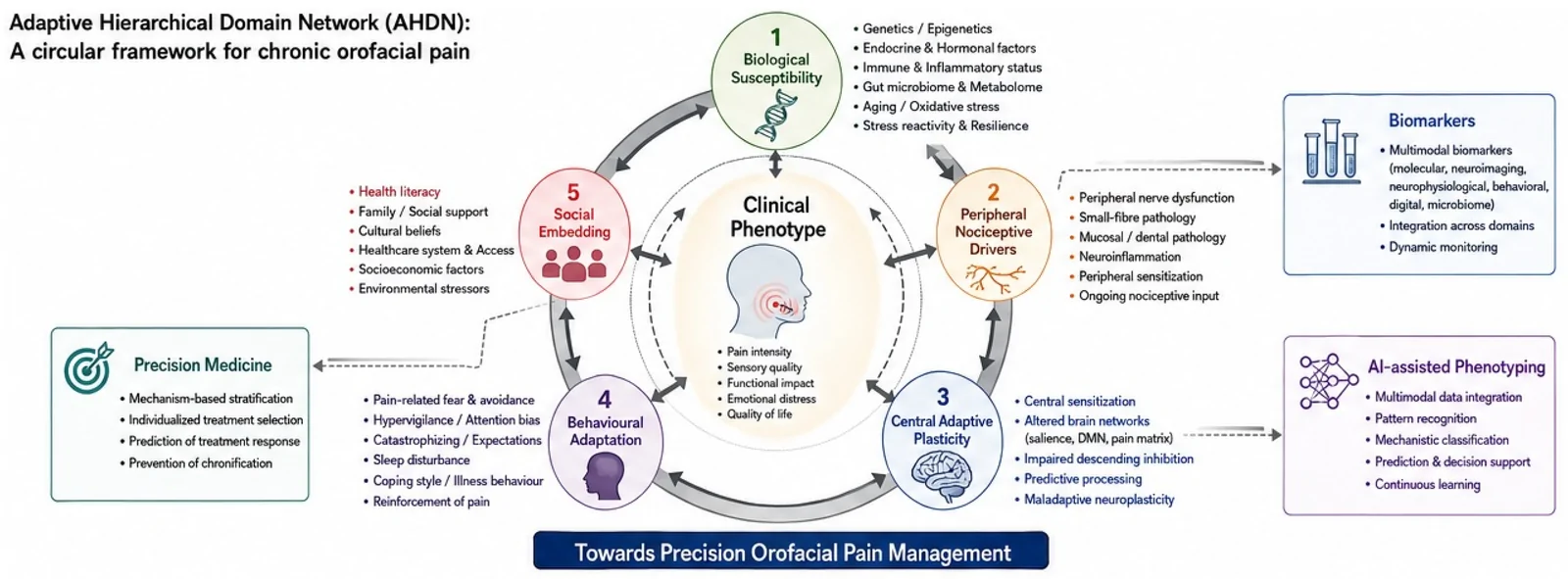
Adaptive Hierarchical Domain Network (AHDN) framework for chronic orofacial pain. Chronic orofacial pain is conceptualized as a dynamic adaptive biological network organized across five hierarchically interacting layers. Biological susceptibility influences peripheral nociceptive drivers, which promote central adaptive plasticity. Behavioral adaptation and social embedding subsequently reinforce or attenuate pain persistence through continuous bidirectional feedback. Clinical phenotypes such as burning mouth syndrome, persistent dentoalveolar pain disorder, persistent idiopathic facial pain, occlusal dysesthesia, and temporomandibular disorders represent different mechanistic configurations within the same adaptive network rather than completely distinct disease entities.

The Adaptive Hierarchical Domain Network (AHDN) is proposed as an integrative clinical framework for understanding chronic orofacial pain as a dynamic biological system rather than as a collection of isolated disease entities (Figure 1). Importantly, the term AHDN does not denote a machine learning algorithm or computational model. Rather, “adaptive,” “hierarchical,” and “network” describe the conceptual organization and dynamic interactions among biological, neural, behavioral, and social mechanisms underlying chronic orofacial pain. Unlike conventional disease classifications, which primarily describe clinical phenotypes, the AHDN framework seeks to explain the biological architecture underlying symptom persistence. It assumes that chronic pain emerges through continuous interactions among multiple biological levels of organization, each influencing and being influenced by the others throughout the course of disease [22].

The term adaptive emphasizes that mechanistic dominance is not static. Biological processes continuously reorganize in response to persistent nociceptive input, neuroplasticity, endocrine changes, behavioral adaptation, therapeutic intervention, and environmental influences. Consequently, patients are expected to move dynamically within the network as disease progresses or treatment modifies underlying mechanisms [23].

The term hierarchical reflects the observation that biological systems operate across multiple organizational levels, ranging from molecular regulation to behavioral adaptation and social context. Rather than functioning independently, these levels interact through reciprocal feedforward and feedback mechanisms [24].

Finally, the term network recognizes that persistent pain is generated not by a single pathological lesion but by interactions among interconnected biological systems [25]. The five-layer structure was developed as a conceptual synthesis of mechanisms repeatedly discussed across the chronic pain, orofacial pain, systems neuroscience, behavioral, and biopsychosocial literature. The layers were not derived from a formal consensus procedure, Delphi process, standardized scoring system, or systematic quantitative synthesis. Rather, they were organized to provide a clinically interpretable representation spanning upstream biological susceptibility, peripheral input, central neural adaptation, behavioral responses, and social context. This organization is therefore intended as a hypothesis-generating framework rather than a definitive taxonomy. Clinical phenotypes therefore emerge as system-level properties of an adaptive biological network.

## 4. Five Hierarchical Layers of the AHDN Framework

The AHDN framework consists of five hierarchically connected layers. Although presented sequentially for conceptual clarity, these layers interact continuously through bidirectional communication and should not be interpreted as independent compartments. The term hierarchical refers primarily to organizational levels rather than a fixed causal or temporal sequence. The layers are arranged from upstream biological susceptibility through peripheral and central mechanisms to behavioral and social embedding for conceptual clarity. However, these levels continuously interact through bidirectional feedback, and changes at a downstream level may influence upstream processes. Thus, the hierarchy should not be interpreted as a rigid one-way causal pathway.

### 4.1. Layer 1: Biological Susceptibility

The first layer represents the biological foundation upon which chronic pain develops. Individual susceptibility is determined by complex interactions among genetic background, epigenetic regulation, endocrine function, immune activity, aging, metabolism, and the gut microbiota [26,27]. These upstream biological systems influence inflammatory responsiveness, neuronal plasticity, stress adaptation, tissue repair, and resilience throughout life. Rather than directly producing pain, biological susceptibility modifies how the nervous system responds to subsequent peripheral injury or environmental stressors. This perspective explains why apparently identical peripheral insults frequently result in markedly different long-term outcomes among different individuals.

### 4.2. Layer 2: Peripheral Nociceptive Drivers

The second layer encompasses peripheral mechanisms capable of initiating persistent nociceptive signaling. Recent studies have demonstrated that many patients previously diagnosed with idiopathic oral pain exhibit subtle pathological abnormalities, including small-fiber neuropathy, trigeminal nerve dysfunction, epithelial neuroinflammation, altered sensory receptor expression, and persistent neuroimmune activation [28]. These abnormalities may generate continuous nociceptive input despite the absence of overt structural pathology. Within the AHDN framework, peripheral pathology is regarded as an initiating component rather than the sole determinant of chronic pain. Its long-term clinical significance depends on interactions with both upstream biological susceptibility and downstream neural adaptation.

### 4.3. Layer 3: Central Adaptive Plasticity

Persistent peripheral input induces adaptive reorganization within the central nervous system. Traditionally, this process has been explained by central sensitization. However, accumulating evidence suggests that chronic pain involves substantially broader alterations affecting distributed brain networks responsible for salience detection, cognitive control, emotional regulation, self-awareness, and sensory prediction [29,30]. The AHDN framework therefore introduces the concept of central adaptive plasticity, which incorporates several complementary mechanisms: central sensitization, altered large-scale functional connectivity, impaired descending pain modulation, predictive processing, and maladaptive sensory learning. Pain gradually evolves from representing external sensory information to becoming an internally generated prediction continuously reinforced by prior experience and expectation. Consequently, chronic pain should be understood as a disorder of adaptive biological prediction rather than simply increased nociceptive transmission.

### 4.4. Layer 4: Behavioral Adaptation

Behavioral responses gradually become integrated into the adaptive pain network. Hypervigilance, fear avoidance, altered oral motor behavior, repeated symptom monitoring, catastrophic interpretation, reassurance seeking, and repeated dental consultations may unintentionally reinforce maladaptive neural predictions [31,32]. These responses are not viewed as evidence that symptoms are psychologically generated. Instead, they represent biologically meaningful adaptations that become maladaptive through repeated reinforcement. Successful behavioral interventions may therefore modify network dynamics by reducing prediction error and facilitating adaptive recalibration.

### 4.5. Layer 5: Social Embedding

The fifth layer recognizes that chronic pain develops within a social environment. Health literacy, clinician–patient communication, family support, cultural beliefs, healthcare accessibility, and socioeconomic circumstances all influence long-term adaptation. Repeated diagnostic uncertainty, inconsistent clinical explanations, unnecessary irreversible procedures, or fragmented healthcare may strengthen maladaptive predictive models [33]. Conversely, effective education, multidisciplinary care, and therapeutic alliance may facilitate recovery by reducing uncertainty and promoting adaptive neural reorganization. Accordingly, social factors should not be regarded merely as external influences but as integral components of the adaptive biological network.

## 5. Dynamic Interactions and Clinical Implementation of the AHDN

### 5.1. Temporal Shifts in Mechanistic Dominance

A distinguishing feature of the AHDN framework is that mechanistic dominance is expected to change throughout disease progression. For example, a patient with burning mouth syndrome may initially exhibit predominantly biological susceptibility and subtle peripheral dysfunction. Persistent nociceptive signaling subsequently promotes central adaptive plasticity, while behavioral adaptation and healthcare experiences progressively reinforce symptom persistence [34].

By contrast, patients with persistent dentoalveolar pain disorder may initially demonstrate stronger peripheral nociceptive drivers following dental intervention, whereas prolonged disease gradually becomes increasingly dependent upon central adaptive plasticity [35]. Similarly, occlusal dysesthesia may emerge primarily through maladaptive predictive processing and behavioral reinforcement despite minimal peripheral pathology [36]. These examples illustrate that identical clinical symptoms may arise through different biological trajectories, while different diagnostic categories may converge upon similar adaptive network states. The AHDN framework therefore emphasizes mechanistic phenotyping rather than disease categorization.

### 5.2. A Five-Stage Practical Workflow for Clinicians

**Figure 2:**
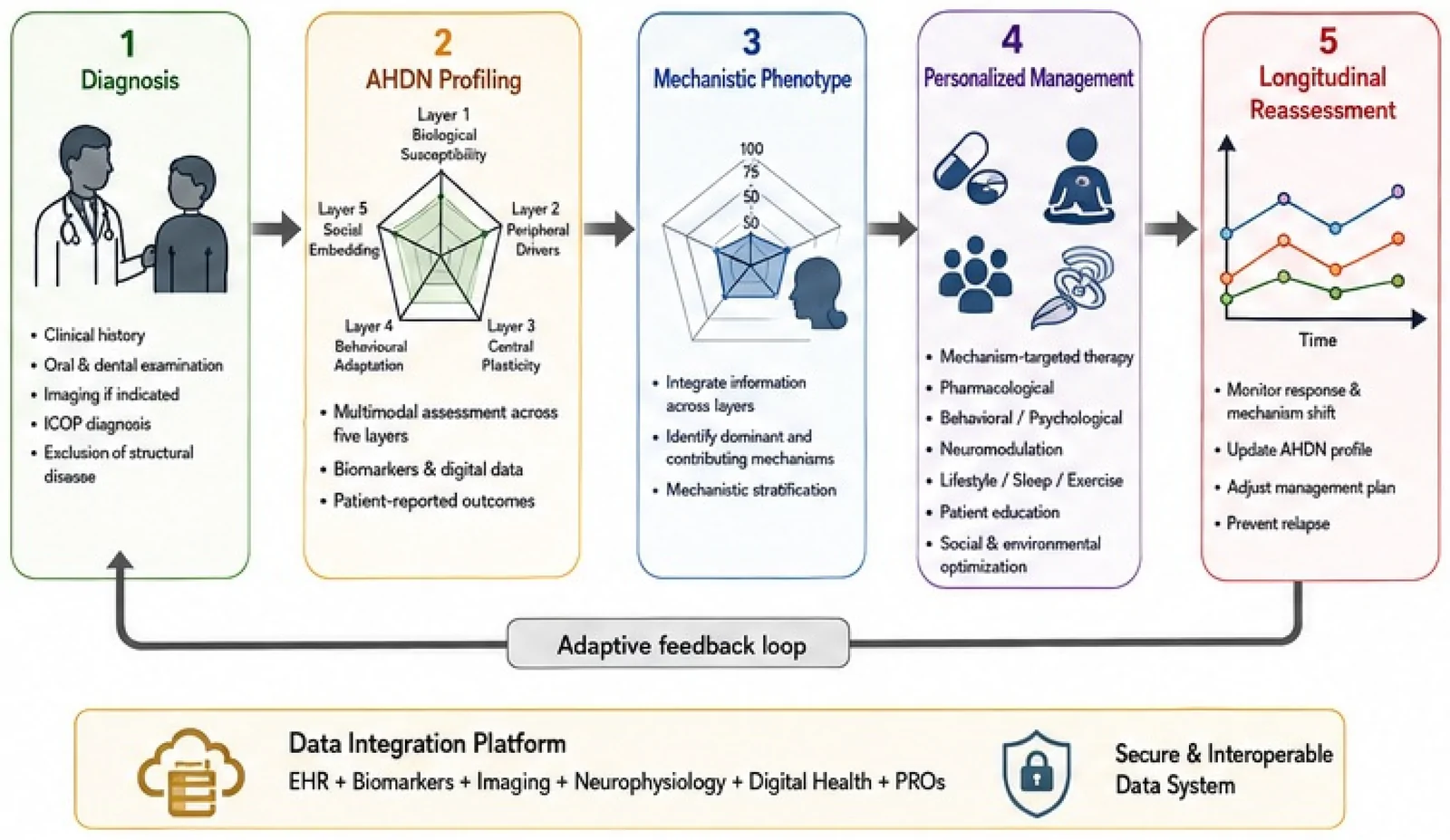
Clinical implementation of the Adaptive Hierarchical Domain Network (AHDN). Conventional diagnosis according to established clinical criteria is followed by systematic evaluation of the five hierarchical layers. The resulting mechanistic phenotype guides personalized intervention and is repeatedly reassessed throughout follow-up, reflecting the adaptive nature of chronic pain. The workflow illustrates the transition from disease-centered diagnosis toward mechanism-based precision management. Note on presentation: The color coding was used to illustrate the workflow of the clinical implementation of the AHDN; therefore, the colors themselves carry no specific meaning.

A conceptual framework has limited clinical value unless it can guide patient evaluation and therapeutic decision-making. One potential value of the AHDN is that it provides a conceptual structure for translating current biological knowledge into future individualized clinical management. Rather than replacing conventional diagnosis, the AHDN framework complements existing disease classifications by identifying the dominant biological mechanisms that sustain persistent pain in each patient. Consequently, patients who share the same diagnosis could potentially be considered for different management strategies according to their mechanistic phenotype rather than their disease label alone, although this approach requires prospective clinical validation (Figure 2).

The proposed clinical workflow consists of five sequential stages:

Stage 1. Conventional Diagnosis: Patients should first undergo a comprehensive clinical evaluation according to established diagnostic criteria, including the ICOP, clinical examination, imaging when indicated, and exclusion of structural disease. Disease classification remains essential because it provides standardized communication and identifies disorders requiring conventional medical or dental treatment [37]. The AHDN framework therefore represents a second layer of interpretation rather than a replacement for established diagnosis.

Stage 2. Mechanistic Profiling: Following diagnosis, clinicians evaluate the relative contribution of each hierarchical layer along a continuum. Rather than asking “What disease does this patient have?” the clinician asks “Which biological mechanisms currently maintain this patient’s symptoms?” Assessment operationalizes clinical evaluation by differentiating routinely accessible clinical tools from investigational or specialized research-stage modalities. Specifically, routine clinical history, standard dental/neurological examination, established validated questionnaires (e.g., for catastrophizing, hypervigilance, and sleep disturbance in Layer 4; social support and health literacy in Layer 5), and standard diagnostic imaging represent immediately applicable routine clinical assessments. Conversely, advanced Quantitative Sensory Testing (QST), mucosal small-fiber neurohistology (Layer 2), functional neuroimaging (fMRI), conditioned pain modulation protocols (Layer 3), specialized systemic/salivary omics, and microbiome profiling (Layer 1) represent investigational or tertiary referral tools. Identifying the “dominant layer” in current practice does not rely on a rigid algorithmic calculation but rather on qualitative clinical synthesis of these converging routine and specialized findings.

Stage 3. Mechanistic Phenotyping: The clinician subsequently integrates information across all five layers to construct an individualized AHDN profile. Patients with identical clinical diagnoses may therefore demonstrate markedly different mechanistic architectures, providing a biologically meaningful explanation for the considerable heterogeneity observed in clinical practice.

Stage 4. Personalized Intervention: Treatment strategies target the dominant mechanisms identified during AHDN profiling. Patients with predominant peripheral abnormalities benefit from interventions directed toward peripheral nociceptive drivers. Conversely, patients demonstrating prominent central adaptive plasticity require centrally acting pharmacological therapies, neuromodulation, behavioral intervention, or multidisciplinary rehabilitation. Interventions for Layer 4 and Layer 5 involve patient education, cognitive behavioral approaches, sleep optimization, and optimization of therapeutic alliance to minimize healthcare-related reinforcement.

Stage 5. Adaptive Reassessment: Patient profiles are expected to change over time. Successful treatment should alter the relative contribution of hierarchical layers (e.g., reducing central adaptive plasticity while improving behavioral adaptation and restoring confidence in normal sensory experiences). Accordingly, mechanistic phenotyping should be repeated during follow-up rather than regarded as a single baseline assessment, keeping the management plan dynamic.

## 6. Clinical Scenarios: Applying the AHDN Framework Across Orofacial Pain Disorders

**Figure 3:**
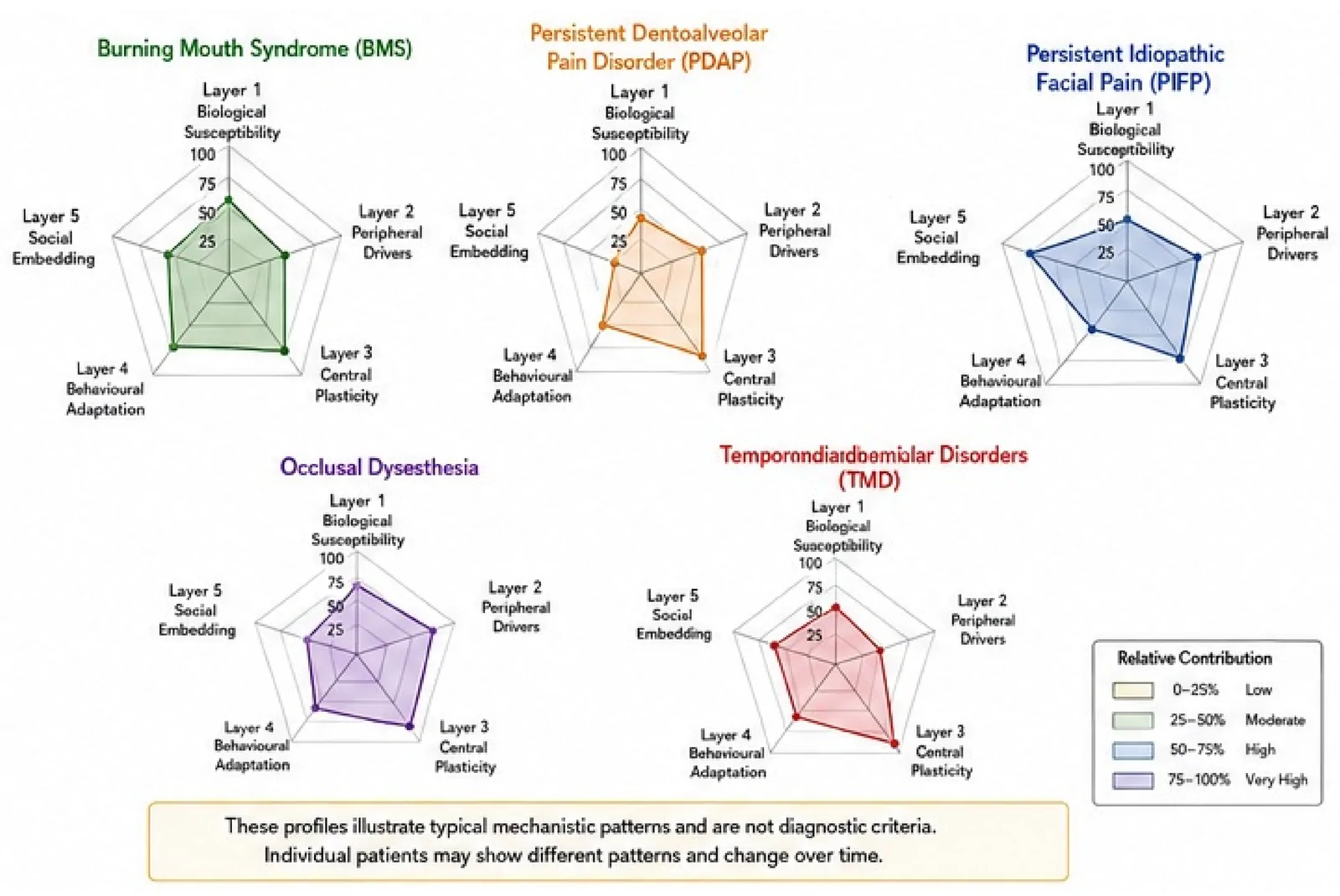
Representative Adaptive Hierarchical Domain Network (AHDN) profiles in major chronic orofacial pain disorders. The relative contribution of each hierarchical layer differs among burning mouth syndrome (BMS), persistent dentoalveolar pain disorder (PDAP), persistent idiopathic facial pain (PIFP), occlusal dysesthesia, and temporomandibular disorders (TMDs). Note on presentation: The numerical radial axes (0 to 100) are purely illustrative, conceptual ordinal representations intended to depict relative mechanistic emphasis (qualitatively corresponding to low, moderate, high, and very high contributions). They are not derived from a quantitative scoring instrument or patient dataset and must not be used as clinical diagnostic scores. The color fills serve solely for visual differentiation among conditions. Individual patient profiles are highly heterogeneous and dynamically evolve over time.

Rather than viewing chronic orofacial pain disorders as discrete disease entities, the AHDN framework interprets them as different mechanistic configurations within the same adaptive biological network. The dominant hierarchical layers vary among patients and may change throughout disease progression. Representative examples are illustrated in Figure 3.

### 6.1. Burning Mouth Syndrome

Burning mouth syndrome (BMS) is among the most heterogeneous chronic pain disorders encountered in oral medicine [38]. Patients often report persistent oral burning despite clinically normal oral mucosa. Multiple biological abnormalities have been reported, including small-fiber neuropathy, altered dopaminergic neurotransmission, impaired descending pain inhibition, hormonal changes, neuroinflammation, and psychological distress [39]. Within the AHDN framework, these findings are interpreted as complementary rather than competing mechanisms. BMS may involve substantial contributions from Layer 1 (biological susceptibility) and Layer 3 (central adaptive plasticity), while peripheral abnormalities may function primarily as initiating rather than sustaining factors. Accordingly, a multimodal management approach may be appropriate for patients in whom multiple mechanisms appear to contribute, namely a combination of pharmacological treatment, patient education, behavioral intervention, and long-term follow-up rather than reliance on a single therapeutic modality.

### 6.2. Persistent Dentoalveolar Pain Disorder

Persistent dentoalveolar pain disorder (PDAP) frequently develops after dental procedures despite complete structural healing [40]. Initially, persistent peripheral nociceptive signaling may dominate the clinical picture. However, prolonged nociceptive input gradually induces adaptive changes within central neural networks, allowing pain to persist even after peripheral pathology has largely resolved. The AHDN framework suggests that some patients may show a progressive transition from Layer 2 dominance toward increasing contributions from Layer 3 and Layer 4. Recognition of this transition has important therapeutic implications because repeated irreversible dental procedures may become progressively less effective as central adaptive plasticity develops. Early identification of such a mechanistic shift could potentially inform treatment planning, although this hypothesis requires prospective evaluation.

### 6.3. Persistent Idiopathic Facial Pain

Persistent idiopathic facial pain (PIFP) remains one of the least understood chronic pain disorders [41]. Many patients demonstrate severe pain despite limited objective peripheral pathology. Within the AHDN framework, this phenotype is interpreted as one in which central adaptive plasticity may represent an important contributor, while behavioral adaptation further stabilizes maladaptive network activity. This perspective may help explain why structural investigations can fail to identify clinically meaningful abnormalities despite persistent symptoms. It also supports multidisciplinary treatment approaches directed toward central mechanisms rather than repeated peripheral interventions.

### 6.4. Occlusal Dysesthesia

Occlusal dysesthesia may provide an informative clinical example of altered predictive processing [42]. Patients experience persistent discomfort or altered bite sensation despite objectively satisfactory occlusion. Repeated occlusal adjustment rarely produces sustained improvement and may occasionally worsen symptoms. The AHDN framework proposes that altered predictive models within cortical sensory networks may contribute to a persistent mismatch between expected and perceived occlusal sensation. Behavioral adaptation, repeated symptom monitoring, and repeated dental treatment may unintentionally reinforce these prediction errors, thereby strengthening maladaptive neural representations. Accordingly, unnecessary irreversible dental procedures may warrant caution when substantial central adaptive plasticity is suspected. Instead, patient education, reassurance, behavioral intervention, and multidisciplinary care become increasingly important.

### 6.5. Temporomandibular Disorders

Temporomandibular disorders (TMDs) comprise a heterogeneous group of conditions with highly variable biological mechanisms. Some patients exhibit predominantly peripheral musculoskeletal pathology, whereas others demonstrate prominent nociplastic features involving altered central pain modulation and psychosocial influences [43]. The AHDN framework accommodates this heterogeneity by allowing different hierarchical layers to predominate in different patients. Consequently, patients sharing identical TMD diagnoses may potentially require different treatment strategies according to their individual mechanistic profiles, although this proposition remains to be clinically validated.

## 7. Mechanism-Based Stratification for Precision Management and Biomarkers

### 7.1. Beyond Disease Labels: Mechanistic Phenotyping

Precision medicine has transformed oncology through molecular characterization of individual tumors. Similar transitions are now occurring in immunology, neurology, and psychiatry, where treatment increasingly depends on biological mechanisms rather than diagnostic labels alone [44]. Chronic orofacial pain has not yet fully benefited from this transition. Current treatment selection remains largely symptom-driven despite growing evidence that apparently similar patients may possess fundamentally different biological mechanisms.

One of the principal messages of the AHDN framework is that disease labels should represent the beginning rather than the end of clinical reasoning. Conventional diagnosis remains indispensable for communication and standardization. However, diagnosis alone provides limited information regarding the biological mechanisms responsible for symptom persistence. The proposed framework shifts clinical decision-making from disease-oriented management toward mechanism-oriented personalized care, providing a practical strategy for implementing precision medicine within oral medicine.

### 7.2. Integrating Multimodal Biomarkers with Targeted Interventions

A key implication of the proposed framework is that biomarkers should be viewed as components of an integrated biological network rather than interpreted in isolation. Crucially, chronic pain is unlikely to be defined by a single biomarker; rather, its biological significance emerges through integration across multiple hierarchical levels. Rather than seeking a single diagnostic biomarker, clinicians may instead integrate multiple complementary biological indicators reflecting different hierarchical layers, which provides greater explanatory power than isolated biomarkers considered independently. Table 1 presents candidate biomarkers and personalized management strategies directly linked with the mechanistic layout of the AHDN across clinical disorders.

## 8. Future Perspectives: Digital Health and Network Medicine

### 8.1. Digital Biomarkers and Artificial Intelligence

Rapid advances in digital health technologies provide new opportunities to implement the AHDN framework in routine clinical practice. Wearable sensors, smartphone-based ecological momentary assessment, sleep monitoring, digital behavioral phenotyping, and remote symptom tracking allow continuous evaluation of patients outside traditional clinical settings. Artificial intelligence and machine learning algorithms may integrate these longitudinal datasets with molecular biomarkers, neuroimaging findings, quantitative sensory testing, and electronic health records to generate individualized mechanistic profiles. Rather than replacing clinical judgment, these computational approaches may support clinicians by identifying biological patterns that are difficult to recognize using conventional assessment alone. Ultimately, AHDN-guided computational phenotyping may facilitate dynamic monitoring of disease progression and treatment response throughout the patient journey. However, computational phenotyping also presents important challenges. Algorithmic bias may arise when training datasets are not sufficiently representative of diverse patient populations, and external validation is required before models can be generalized across clinical settings. Data privacy, governance, and security are particularly important when longitudinal behavioral, physiological, and electronic health record data are integrated. In addition, limited explainability may complicate clinical interpretation and accountability. Unequal access to digital technologies may further widen existing healthcare disparities. Therefore, AI-assisted phenotyping should currently be regarded as a potential decision-support approach rather than a substitute for clinical judgment, and its clinical implementation will require rigorous validation, transparency, appropriate governance, and equitable access.

### 8.2. Biomarker Discovery Through Network Medicine

Network medicine provides another important future direction. Conventional biomarker research often evaluates isolated molecular pathways independently. However, chronic pain is unlikely to result from abnormalities within a single biological system. The AHDN framework instead proposes that clinically meaningful biomarkers will emerge through the integration of multiple biological domains. Future studies may therefore combine genomics, epigenomics, transcriptomics, proteomics, metabolomics, microbiome profiling, neuroimaging, and digital biomarkers within unified computational models capable of describing adaptive biological networks. Such multidimensional approaches may improve patient stratification while accelerating biomarker discovery and therapeutic development.

## 9. Implications for Clinical Practice

The principal contribution of the AHDN framework is not the introduction of new diagnostic categories but the provision of a structured method for organizing existing knowledge. Current classifications remain indispensable for diagnosis and communication. However, patients increasingly require individualized management based on biological mechanisms rather than disease labels alone. By integrating biological susceptibility, peripheral pathology, central adaptive plasticity, behavioral adaptation, and social embedding within a single framework, AHDN provides clinicians with a practical strategy for interpreting complex clinical presentations. This approach encourages multidisciplinary collaboration among oral medicine specialists, neurologists, psychiatrists, psychologists, pain physicians, and primary care clinicians. Most importantly, it supports a transition from reactive symptom management toward proactive mechanism-based precision care.

## 10. Limitations

Several limitations of the proposed framework should be acknowledged. First, the AHDN framework is a conceptual, hypothesis-generating model that has not yet undergone prospective clinical validation. Second, the framework was developed as a conceptual synthesis of existing literature and expert interpretation rather than through a formal consensus or Delphi process, and patients were not directly involved in its development. Third, no standardized scoring criteria currently exist for determining the relative contribution of individual AHDN layers. Accordingly, the numerical representations shown in Figure 3 should not be interpreted as validated clinical scores. Fourth, the five hierarchical layers should not be interpreted as rigid or mutually exclusive categories. Biological adaptation is characterized by continuous bidirectional interactions, and the relative contribution of each layer is expected to vary among individuals and throughout the course of disease. Fifth, several candidate biomarkers discussed in this article remain investigational and are not yet suitable for routine clinical implementation. Their inclusion is intended to illustrate potential future directions rather than current standards of care. Finally, prospective studies will be required to determine whether AHDN-based mechanistic phenotyping improves patient stratification, treatment selection, or clinical outcomes.

## 11. Conclusions

Chronic orofacial pain remains one of the most complex and heterogeneous conditions encountered in oral medicine. Although recent diagnostic classifications have improved clinical consistency, substantial mechanistic heterogeneity continues to limit precision diagnosis and individualized treatment. The Adaptive Hierarchical Domain Network (AHDN) framework provides an integrative clinical model that complements existing diagnostic systems by organizing current biological knowledge into five hierarchically interacting domains: biological susceptibility, peripheral nociceptive drivers, central adaptive plasticity, behavioral adaptation, and social embedding. Rather than replacing established diagnostic categories, the framework encourages clinicians to identify the dominant mechanisms sustaining pain in individual patients and to tailor management accordingly.

By integrating concepts from systems biology, network neuroscience, predictive processing, precision medicine, and network medicine, AHDN provides a hypothesis-generating conceptual structure for future biomarker discovery, individualized therapeutic strategies, and translational research. As multimodal biomarkers, digital health technologies, and artificial intelligence continue to evolve, mechanism-based phenotyping may become increasingly feasible. However, the AHDN framework should currently be regarded as a conceptual model rather than a validated clinical roadmap. Prospective validation, standardized assessment methods, and evaluation of clinical utility will be necessary before AHDN-based mechanistic phenotyping can be considered for routine clinical implementation.

## Figures and Tables

**Table 1:** Proposed relationship between AHDN mechanisms, candidate biomarkers, and personalized management strategies.

Clinical Disorder	Dominant AHDN Layers	Candidate Biomarkers	Evidence Status and Clinical Readiness	Scientific Implications	Potential Personalized Management
Burning Mouth Syndrome	Layer 1 + Layer 3	Salivary biomarkers, endocrine profile, QST, resting-state fMRI	Clinically available assessments; candidate biological biomarkers remain investigational	Predominantly central adaptive phenotype	Centrally acting pharmacotherapy, behavioral therapy, neuromodulation, patient education
Persistent Dentoalveolar Pain Disorder	Layer 2 → Layer 3	Small-fiber assessment, QST, trigeminal neurophysiology	QST and peripheral sensory assessment are clinically informative in selected settings; mechanistic biomarkers remain investigational	Progressive transition from peripheral to central mechanisms	Early mechanism-based intervention, prevention of chronicity
Persistent Idiopathic Facial Pain	Layer 3 + Layer 4	Functional connectivity, conditioned pain modulation, digital phenotyping	Functional and sensory assessments are available in research/selected clinical settings; digital phenotyping remains investigational	Predominantly nociplastic phenotype	Multidisciplinary pain management, cognitive behavioral therapy
Occlusal Dysesthesia	Layer 3 + Layer 4 + Layer 5	Sensory integration assessment, behavioral profiling	Clinical sensory and behavioral assessment is available; mechanistic profiling remains investigational	Predictive processing dysfunction	Patient education, behavioral intervention, avoidance of irreversible occlusal procedures
Temporomandibular Disorders	Variable	MRI, QST, psychosocial assessment	MRI/QST and psychosocial assessment may be clinically available depending on setting; integrated mechanistic profiling remains investigational	Mixed mechanistic phenotype	Individualized multimodal management

## Data Availability

The original contributions presented in this study are included in the article. Further inquiries can be directed to the corresponding author.

## Abbreviations

The following abbreviations are used in this manuscript:

AHDN	Adaptive Hierarchical Domain Network
BMS	burning mouth syndrome
ICOP	International Classification of Orofacial Pain
PDAP	persistent dentoalveolar pain
PIFP	persistent idiopathic facial pain
TMDs	temporomandibular disorders
